# Factors Affecting In-stent Restenosis in Patients Undergoing Percutaneous Coronary Angioplasty

**DOI:** 10.22086/gmj.v0i0.961

**Published:** 2018-06-15

**Authors:** Mostafa Sajadian, Ladan Alizadeh, Mahmoud Ganjifard, Armin Mardani, Mohammad Ali Ansari, Homa Falsoleiman

**Affiliations:** ^1^Atherosclerosis Prevention Research Center, Department of Cardiovascular Diseases, Faculty of Medicine, Mashhad University of Medical Sciences, Iran; ^2^Department of Anesthesiology, Faculty of Medicine, Birjand University of Medical Sciences, Iran

**Keywords:** Angioplasty, Stents, Coronary Stenosis, In-Stent Restenosis

## Abstract

Percutaneous coronary angioplasty (PCI) and stent implantation are the most common therapeutic strategies for coronary artery stenosis; however, in-stent restenosis (ISR) is one of its important challenges. Although in some studies, coronary artery disease (CAD) factors are deemed to be the causes of ISR, in some others, the relationship between CAD factors and ISR are not observed. Over the past ten years, there has been no review article on factors affecting the ISR. This article aimed to review the possible factors affecting ISR in patients undergoing PCI. This narrative review study was conducted on PubMed, Web of Science, Scopus, and Google Scholar databases between 1 January 1990 and 30 July 2017. After initial screening of 1728 retrieved articles, 1401 articles were excluded to due irrelevancy to the review; and finally, 39 papers were selected for data collection. Our study results showed that the site and length of implanted stent, hypertension and diabetes are the most probable factors affecting ISR. Further studies are required for evaluation of the effect of other possible risk factors such as genetic sequencing, obesity, chronic infections and hemoglobin A1C levels.

## Introduction

Coronary artery disease (CAD), in particular, acute coronary syndrome, and stable angina are some of the most common causes of death in the world [1]. Stent implantation during percutaneous coronary angioplasty (PCI) is one of the leading non-therapeutic treatments for coronary artery stenosis [1-2]. Although stenting is used among over 70% of PCI case, this action is not associated always with positive results. Therefore, the occurrence of in-stent restenosis (ISR) has become a significant challenge following some actions [2-4] The incidence rate of ISR has already been reported between 3.3 and 41%; for example, it is estimated that annually about 150 thousand people in America are suffering from this complication [1-4].

Endothelial dysfunction, smooth muscle proliferation, and inflammation have been identified as the primary mechanisms of ISR after stent implantation [5-8]. Apart from vascular factors, other risk factors such as older age, gender, hypertension, hyperlipidemia, diabetes mellitus, and smoking have also been mentioned as contributing factors in the incidence of restenosis. However, the results are different in various studies, and the role of these variables has not been established definitively [3, 5, 9-12]. On the other hand, the type of stent used, diameter and length of the stent, the artery with stent implantation as well as the duration of stent implantation have also been named as effective factors in the ISR [4, 13, 14]. Over the past ten years, there has been no review article on factors affecting the ISR. Therefore, it seems that a new review study could offer a better view to specialists toward the possible influential factors in the ISR. This aim of this study was to review the possible coronary risk factors affecting ISR in patients undergoing PCI.

##  Search Strategies

This narrative review study was conducted on extractions of original studies, editorials, and reviews using the combination of keywords “Cardiovascular Diseases”, “acute coronary syndrome”, “Stent implantation”, “percutaneous coronary angioplasty”, “angiography”, “stent”, “stent stenosis”, “restenosis”, “stenosis”, “in-stent restenosis”, “hypertension”, “hyperlipidemia”, “diabetes mellitus” and “smoking”. Databases including PubMed, Web of Science, Scopus and Google Scholar within a time limitation between 1 January 1990 and 30 July 2017 were investigated. Articles with both Persian and English languages were included. There were no restrictions on the type of study design and the method of assessing in-stent restenosis. References of all included studies were examined to identify any other potentially relevant articles. A total of 1728 articles retrieved from the searches were firstly screened based on the title and abstract, by a single reviewer. Initial screening led to the exclusion of 1401 articles irrelevant to the review. The remaining 327 papers were retrieved of which 39 were selected for data collection.

### ISR and Gender

Very few studies have compared the incidence rate of ISR between men and women. Robert *et al*. [15] reported that the incidence rate of ISR was slightly higher in women than in men. According to a review article, women are more likely at risk of ISR than in men, though this difference has not been reported significant [16]. In studies conducted by van Domburg el al. and Popma *et al.*, female and male genders were respectively recognized as predictors of ISR, while Heidland *et al*. reported that gender was not significantly associated with increased risk of ISR [17-19]. In the study of Bausters *et al.*, the ISR rate after six months follow-up showed no statistically significant differences in men and women, respectively about 26% and 27% [5]. Finally, it seems that the majority of studies course refers to the lack of correlation between the ISR and gender; and definitive conclusions cannot yet be made about the relationship between gender and ISR.

### ISR and Age

Pourmoghaddas *et al*. showed that the restenosis rate increased with age so that the highest rate of restenosis was observed in the age group of 50-59 years [20]. Also, old age in the studies of Popma and Van Domburg has been raised as an independent predictor of restenosis after angioplasty [17, 18]. However, there are conflicting results so that Heidland and Gurlek *et al*. showed that age is not involved in the increased risk of restenosis [16, 19].

In a meta-analysis published in 1995, no significant differences were observed for age between groups with and without ISR [16]. According to studies, it seems that old age cannot be considered a definitive cause for the occurrence of ISR, though its role is apparently stronger compared to gender. Further cohort studies are needed to confirm the specific role.

### ISR and Smoking

In some studies, smoking has been declared an independent risk factor to predict the restenosis [21]; however, studies such as Gurlek and Kotamaki *et al*. showed that smoking does not increase the restenosis [16, 22]. In the study of Pourmoghaddas, the rate of ISR was reported respectively 29.6% and 17.2% in smokers and non-smokers, representing a significant increase in restenosis among smokers [20]. In a study of Taira *et al*. carried out on 1432 patients undergoing stent implantation in two centers, the results showed that smokers more than non-smokers were significantly suffering from ISR [23]. However, due to the role of smoking in causing cell proliferation and thereby an increased risk of stent stenosis, further studies seem to be reasonable in relation to the role of smoking in the incidence of restenosis [24].

### ISR and Diabetes

Among the risk factors raised for coronary, diabetes is a risk factor with high predicting risk for restenosis [25, 26]. In a study conducted on 589 patients undergoing stent implantation, the results of 12-month follow-up showed that diabetes has a significant relationship with the occurrence of ISR [26]. According to a study conducted by Kastrati *et al.*, diabetes causes an increased risk of restenosis with an odds ratio (OR) of 1.86 [27]. In a report of Lee *et al.*, diabetes was associated with a higher incidence rate of ISR [28]. On the other hand, in a study by Lau *et al.*, the role of diabetes has been introduced as the most potent predictor of ISR [29]. In a study carried out on the Iranian race, diabetes increases the ISR from 23.5% to 50% [19]. To confirm these findings, the results of meta-analysis revealed that diabetic patients compared to those without diabetes have significantly higher OR to develop ISR (10.8% vs. 4.4%) [16]. Due to the results obtained in different studies, it seems that diabetes can be regarded as the most definitive risk factor for ISR.

### ISR and Hypertension

Most studies have reported a significant correlation between the ISR and hypertension. Hasani *et al*. also found a significant relationship between high blood pressure and ISR [30]. In another study, the rate of restenosis in patients with hypertension compared to healthy individuals was reported respectively 37.5% and 47%, indicating an increased rate of restenosis in the group with hypertension [20].

Moustapha *et al*. also showed that 76% of patients with restenosis had high blood pressure [20]. To support the findings of published original articles, a meta-analysis conducted in 1995 on previous studies also showed that high blood pressure was significantly associated with ISR [16].

### ISR and Dyslipidemia

Dyslipidemia is one of the significant risk factors for atherosclerosis, whereas its role has not been decisive in predicting ISR and there are differences of opinion in this regard. Gurlek indicated that dyslipidemia is associated with ISR [16]. During the study of Violaris *et al.*, there was no association between cholesterol levels and ISR [31]. However, some studies have shown that high levels of serum cholesterol are associated with increased restenosis [3, 12]. In a study by Kishi *et al.*, dyslipidemia has been shown to be associated with the occurrence of ISR [32]. Also in the study by Fishman *et al*., dyslipidemia has been mentioned as one of the predictors of ISR [12].

In another study, the incidence of ISR was respectively reported to be 34.3% and 18% in patients with high levels of serum cholesterol in patients without high cholesterol [20]. It seems that relationship of ISR and dyslipidemia is not a verifiable certainty and further studies should be done to investigate the relationship between these two variables.

### ISR and Stent Implantation Site

Some studies have shown that the rate of restenosis is more common in left anterior descending (LAD) in distal or proximal artery lesions [33, 34]. In the study of Pourmoghaddas, restenosis in the proximal segments lesions was significantly more than middle and distal lesions [20]. Another study found that the highest incidence rate of ISR was respectively in stents implanted in a right coronary artery (RCA), in LAD, and left circumflex (LCX) arteries [20]. In general, concerning relationship of ISR and stenosis site and stent implantation, it should be noted that although the majority of studies have indicated higher levels of ISR in LAD, distal and proximal vessels, but number of studies apparently is still deficient to generalize the results and further studies are needed to confirm a definitive link.

### ISR and Stent Length

Based on the results of the studies, it appears that the stent length can act as an essential factor in the occurrence of ISR so that the stent length over 10 mm has a higher odds ratio of restenosis [35, 36]. In a study by Dietz *et al.*, patients with an average length of about 9 mm stents compared to those with an average length of about 16 mm stents experienced significantly less ISR [37]. However, this relationship needs to be further investigated in future studies, because it is still a meager number of studies on this issue [38].

### ISR and Inflammatory Responses

Intima and media layers damages after coronary and peripheral angioplasty cause an inflammatory response [8]. Chronic artery inflammation plays a role in the development of restenosis after balloon angioplasty and stent implantation [7, 39]. Therefore, the measurement of acute-phase serum parameters is a sensitive method for quantitative analysis of vessel inflammation process [39]. CRP is an acute phase protein, which activates the complement system, facilitates the adhesion of neutrophils and is an independent risk factor for atherosclerosis [40]. However, despite extensive studies, the relationship between ISR and levels of inflammatory factors is still contradictory in different studies. Study on 40 patients undergoing stent implantation demonstrated that high level of C-reactive protein (CRP) 96 hours after stent implantation is associated with a high risk of restenosis [40].

Zurakowski *et al*. also showed that higher level of inflammatory markers, CPR and interleukin-6, for six months after stent implantation is associated with restenosis after stent implantation [41]. Also, the mean level of high-density lipoprotein-c (HDL-c) was significantly lower in patients with ISR than the group with an open stent. It seems that the higher level of HDL-c after stent implantation has a protective effect against ISR. This finding may be due to anti-inflammatory effects of HDL-c [42]. On the other hand, and conflicting results, no association was seen among the levels of CRP, interleukin-6 and Amyloid A Serum (SAA) with ISR in patients treated for elective stent implantation [43]. In the study of Dibra *et al.*, ISR was observed at similar rates in groups with normal and high levels of CRP, 24% and 25% respectively [44]. By analyzing the obtained findings, it seems that the lack of focus of various studies on a particular type of inflammatory markers and dissimilarity in the time of testing cause dispersion in the results and inability to correct conclusions in conjunction with the correlation between the inflammatory response and ISR.

## Discussion

The incidence rate of ISR in people undergoing repeated angiography has been reported between 3.3 and 41% [4, 9, 10, 27]. It seems that the main reason for the difference in the incidence rate of restenosis in various studies is the difference in duration of follow-up so that this time in different studies has been varied between 6 months and 15 years [45]. Another issue causing differences among the groups is the difference in the selection of statistical samples from a primary population of patients. Some studies have been conducted on all primary patients, and others have examined solely the patients entered into the study based on cardiac symptoms or specific indications with angiography criteria. Apart from the low statistical sample size that is always one of the possible reasons for the failure to find a significant relationship among variables, one issue that has not been considered in most studies is discussion on impact of other factors such as the type of stent, skills of cardiologists and medicinal purposes on the incidence of ISR.In some studies, one person had performed all stent implantation, but the majority of studies have not been mentioned in this issue. Therefore, it seems that ISR would be the result of work quality of cardiologist in stent implantation, which this should be considered in future studies. Type of stent is also another factor directly influencing the incidence of ISR. However, the type of stent and its relationship with the incidence of ISR have not been evaluated in most studies published. Probably, one of the possible causes of the differences in the results of studies is related to different types of stent. This relationship might be able to act even stronger in a statistical model of the impact of known risk factors for ISR. The next issue that can lead to a difference in the results of various studies is the variation in the exposure duration of patients to each cardiovascular risk factor, such as hypertension, dyslipidemia, diabetes mellitus, smoking and ways to control and treatment. However, besides all the risk factors proposed so far, a paper published by Thanh *et al*. in 2012 made new horizons regarding factors affecting artery stenosis. It seems that anatomical and geometrical changes in arteries are some other effective cases of intravascular stenosis [46].

## Conclusion

Based on the results of this study, it seems that among the known risk factors for

coronary artery disease, the site and length of stent implanted as well as inflammatory markers on the ISR, hypertension, and diabetes are two factors that make them more likely to discuss about the ISR. Although other factors such as smoking, older age, and length of the stent also are in the next places of importance in the incidence of ISR, further studies seem essential in this area. However, other important risk factors such as genetic sequencing, obesity, chronic infections and hemoglobin A1C levels were not investigated in relationship with ISR in this review article, while risk factors for ISR in many studies have been only limited to these new risk factors.

## Conflicts of Interest

There are no conflicts of interest.
